# Effects of Visual and Acoustic Distraction on Driving Behavior and EEG in Young and Older Car Drivers: A Driving Simulation Study

**DOI:** 10.3389/fnagi.2018.00420

**Published:** 2018-12-18

**Authors:** Melanie Karthaus, Edmund Wascher, Stephan Getzmann

**Affiliations:** IfADo – Leibniz Research Centre for Working Environment and Human Factors, Dortmund, Germany

**Keywords:** aging, driving, distraction, cognitive control, EEG

## Abstract

Driving safety depends on the drivers’ attentional focus on the driving task. Especially in complex situations, distraction due to secondary stimuli can impair driving performance. The inhibition of distractors or inadequate prepotent responses to irrelevant stimuli requires cognitive control, which is assumed to be reduced with increasing age. The present EEG study investigated the effects of secondary acoustic and visual stimuli on driving performance of younger and older car drivers in a driving simulator task. The participants had to respond to brake lights of a preceding car under different distraction conditions and with varying task difficulties. Overall, the anticipation of high demanding tasks affected braking response behavior in young and especially in older adults, who showed reduced cognitive control to task-relevant braking stimuli, as reflected by a smaller P3b. In a more easy (perception only) task, simultaneously presented acoustic stimuli accelerated braking response times (RTs) in young and older adults, which was associated with a pronounced P2. In contrast, secondary visual stimuli increased braking RTs in older adults, associated with a reduced P3b. In a more difficult (discrimination) task, braking response behavior was impaired by the presence of secondary acoustic and visual stimuli in young and older drivers. Braking RT increased (and the P3b decreased), especially when the responses to the secondary stimuli had to be suppressed. This negative effect was more pronounced with visual secondary stimuli, and especially so in the older group. In sum, the results suggest an impaired resistance to distractor interference and a reduced inhibition of prepotent responses in older drivers. This was most pronounced when the processing of task-relevant and irrelevant stimuli engage the same mental resources, for example, by sharing the same stimulus modality.

## Introduction

Driver distraction is one of the most important causes of road traffic accidents in western countries (e.g., National Center for Statistics and Analysis, 2018). Typical examples for activities that divert attention from driving are conversations with other passengers (Dingus et al., 2016) and situations in which the driver looks away from the road, e.g., when using cell phones, reaching for objects, operating air-conditioning or infotainment systems. It is unclear which types of distraction cause the greatest decline in driving performance. According to the theory of multiple resources (Wickens, 2002, 2008), interference of parallel stimulus processing or activities should be higher (and thus inhibition of distracting stimuli or activities more demanding) when these stimuli or activities are very similar or use the same mental resources. Given that most of the driving-related information is visual, secondary visual stimuli should thus be more difficult to inhibit than additional acoustic stimuli (e.g., Wickens and Seppelt, 2002; Sodnik et al., 2008). However, there are also large distraction effects by acoustic-verbal interactions with in-vehicle-information-systems (Strayer et al., 2016) and by talking to other passengers or on the cell phone (for a recent review, see Caird et al., 2018). These negative effects could be due to the relatively high complexity of this kind of distraction (Patten et al., 2004; Schweizer et al., 2013). According to Wickens (2002), the benefit of separate resources is diluted when one task requires so many cognitive resources that “nothing is left” for the second task. This concept of resource allocation could explain the extensive distraction effects of talking or calling, even though these tasks access quite different resources than the driving task.

The latter assumption is in line with the cognitive control hypothesis, postulating a selective impairment as a function of cognitive workload. Accordingly, driving subtasks that require cognitive control are affected by workload, while automatized processes are not (Engström et al., 2017). Typical examples in the driving context are classical dual task situations, in which the driver has to decide quickly whether an external stimulus is relevant for driving and has to be responded to (e.g., the flashing up of a brake light of a preceding car), or whether it is irrelevant (e.g., a message on the cell phone) and can be ignored or even has to be suppressed in a critical driving situation.

The underlying processes of cognitive control and – in particular – inhibition are neurologically associated with the prefrontal cortex and prone to age-related changes (e.g., Zelazo et al., 2004). A number of studies observed an age-related inhibition deficit (e.g., Persad et al., 2002; for a recent meta-analysis, see Rey-Mermet and Gade, 2017), especially in tasks with high executive inhibitory control demands (e.g., Andrés et al., 2008). Some authors even attribute the majority of age-related declines in executive functions to a general inhibition deficit (Hasher and Zacks, 1988). Considering its importance for traffic safety, the question occurs to what extent age-related declines in cognitive control play a role for driving abilities of the elderly. There is empirical evidence that older drivers have difficulties especially in complex driving situations (e.g., on multilane crossroads and left turns; Karthaus and Falkenstein, 2016), where cognitive factors play an important role. These include various cognitive control functions such as monitoring one’s own actions, inhibiting inadequate reactions and suppressing distraction, as well as controlling attention (Salthouse, 1996; Verhaeghen and Salthouse, 1997). Further cognitive functions that are subject to age-related changes are the flexible alternation between different tasks and response requirements (Kray and Lindenberger, 2000), which can be a special problem in the traffic context when routines like driving on well-practiced routes are interrupted. Several studies point to specific problems in inhibitory control, indicated by the fact that older drivers seem to process irrelevant stimuli as intensively as relevant stimuli (Hahn et al., 2011), especially in driving situations with many (concurring) distractions (Charness and Bosman, 1992).

The present study used a simulated car driving scenario to investigate the interplay of attention to traffic-related information and inhibition of driving-irrelevant distracting stimuli in younger and older drivers. The drivers had to respond to critical events, while driving under different distraction conditions. Distraction was operationalized in form of additional stimuli that were presented either alone or in combination with the critical event. Task workload was manipulated by varying the amount to which these secondary stimuli had to be processed: In the easy (perception only) condition the participants could ignore the secondary stimuli, while in the more difficult (discrimination) condition they had to attend and respond to the secondary stimuli. It was hypothesized that older drivers have more difficulties than young drivers, especially in the more complex discrimination condition. This should become evident in larger response times (RTs) to the critical event and higher error rates. To investigate the role of modality, the secondary stimuli were either presented acoustically or visually. According to the theory of multiple resources (Wickens, 2002, 2008), visual stimuli should have a greater distracting effect than acoustic stimuli.

In addition to behavioral performance, electrophysiological measures were analyzed to better understand the mechanisms behind the interaction of cognitive control demands and driving performance. The analyses focused primarily on the P3b component of the event-related potentials (ERPs) that is associated with controlled cognitive attentional and stimulus evaluation processes (for review, see Polich, 2007). The P3b is of special interest here, as inhibition deficits in the driving context should be primarily caused by late cognitive processing (for a review about inhibition and ERPs, see Pires et al., 2014). The P3b component has been used to study age-related inhibition processes in a variety of previous tasks and with auditory and/or visual stimuli. Studies employing Go/NoGo-paradigms, in which participants had to respond to target (Go) stimuli and to ignore distracting (NoGo) stimuli, found larger P3b amplitudes and longer P3b latency in NoGo-trials (e.g., Horvath et al., 2009), and smaller P3 amplitudes in Go-trials in older compared to younger adults (Hahn et al., 2011). In the present driving context, we expected that reduced cognitive control in the more difficult task condition should be reflected by smaller and delayed P3b amplitudes, relative to the easy task. In line with the theory of multiple resources, this distraction effect should be more pronounced with visual than acoustic stimuli, and especially so in older drivers.

In addition to the parietal P3b, the fronto-central P2 has been analyzed to test whether earlier processes also play a role for cognitive control of distracting stimuli in the driving context. The P2 is associated with processes of sensory gating, selective attention, feature detection and other early stages of stimulus encoding (O’Donnell et al., 1997; Crowley and Colrain, 2004; Potts, 2004; Lijffijt et al., 2009) and has also been related to protective mechanisms against interference from irrelevant stimuli (García-Larrea et al., 1992; Benikos et al., 2013). Accordingly, we expected that sensory features of the distracting stimuli (i.e., modality) should affect P2, whereas later aspects (i.e., whether the distracting stimuli have to be responded to or not) should affect P3b. Given that age-related slowing of executive functions is also assumed to play a role for cognitive control (Pires et al., 2014), we analyzed both amplitudes and latencies of P3b and P2.

Taken together, the present study used behavioral and neurocognitive measures to investigate the relationship between cognitive control demands, mental workload, and distraction in younger and older car drivers, varying task difficulty and modality of distraction in a simulated driving scenario.

## Materials and Methods

### Participants

A total of 40 participants took part in the experiment, with 20 younger (19–26 years; *M* = 22.9, *SD* = 1.8; 10 female) and 20 older (55–65 years; *M* = 59.6, *SD* = 3.2; 10 female) active car drivers. The young participants were recruited from local colleges and via social media, while the older participants were recruited via flyers distributed in the institute and at events for elderly people. None of them reported any history of neurological or psychiatric disorder or were taking any drugs that may affect the central nervous system. All reported normal or corrected to normal vision and hearing. There were no significant differences between young (*M* = 27.50, *SD* = 2.16) and older (*M* = 26.80, *SD* = 2.48) adults (*p* > 0.05) in the Montreal Cognitive Assessment (MoCA; Nasreddine et al., 2005). Moreover, the two age groups were comparable in several (demographic) variables that might be associated with driving performance or distractibility (see Table 1). All subjects provided informed written consent prior to entering the experiment. They received 40 € for their participation in the experiment. The study was approved by the local ethics committee of the Leibniz Research Centre for Working Environment and Human Factors.

**Table 1:** Comparison of the two age groups in driving relevant variables (assessed by a questionnaire about driving behavior).

	Young	Old	t or X^2^	*p*
Annual mileage	*M* = 9,083 *SD* = 6,353	*M* = 9,175 *SD* = 5,427	*t*(36) = -0.96	*p* = 0.962
Age of acquisition of driver’s license	*M* = 17.40 *SD* = 1.23	*M* = 21.00 *SD* = 6.59	*t*(38) = -2.40	*p* = 0.026
Duration of driving experience in years	*M* = 5.45 *SD* = 2.19	*M* = 38.55 *SD* = 7.33	*t*(38) = -19.35	*p* < 0.001
**Percentage of participants, who…**				
… drive a car daily or at least several times per week	90%	100%	X^2^(1, *N* = 40) = 2.11	*p* = 0.147
… use navigation systems	55%	75%	X^2^(1, *N* = 40) = 1.76	*p* = 0.185
… had one or more crashes in the last 3 years	15 %	15%	X^2^(1, *N* = 40) = 0.00	*p* = 1.000
… feel easily distracted (e.g., in cities)	20 %	5%	X^2^(1, *N* = 40) = 2.06	*p* = 0.151
… rate their driving abilities as better or at least as good as driving abilities of older/younger drivers	70 %	85%	X^2^(1, *N* = 40) = 1.29	*p* = 0.256
… rate their driving abilities as better or at least as good as driving abilities of other drivers of the same age	90 %	70%	X^2^(1, *N* = 40) = 2.50	*p* = 0.114
… enjoy car driving	95 %	80%	X^2^(1, *N* = 39) = 1.89	*p* = 0.169


### Task and Procedure

Before the experiment started, participants filled out a questionnaire about their driving history, driving habits and attitudes toward driving. At the beginning of the test session, they completed the MoCA (Nasreddine et al., 2005) to indicate possible cognitive deficits. Afterward the driving simulation in a static driving simulator (ST Sim, St Software B.V. Groningen, Netherlands) started. To become familiar with the driving simulation task, the participants started with a short practice block (about 5 min). Before starting the experimental blocks, it was ensured that the participants were able to identify the words as city or country names.

Participants had to drive a virtual car on a straight two-lane road through monotonous grassland. While following another car at a constant distance of 15 m with the fixed speed of 31 mph (50 km/h), they were instructed to keep the car on the middle of the (right) lane as accurately as possible. Different road slopes forced the car continuously from the left to the right lateral road position and back, simulating crosswind. Since the strength and direction of the crosswind varied in accordance to a complex signal of eight different superimposed and phase-delayed sine waves (1/25.6, 1/17, 1/12.8, 1/10.2, 1/8.6, 1/7.2, 1/6.4, and 1/5.6 Hz), participants were not able to predict the upcoming crosswind (see also Hahn et al., 2011; Wascher et al., 2016).

**FIGURE 1:**
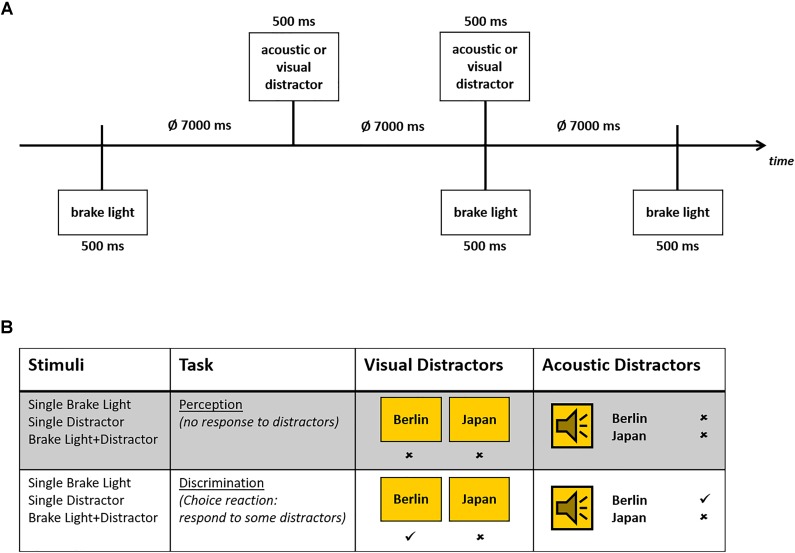
**(A)** Timing of the stimulus presentation in the driving task, in which brake lights and secondary stimuli were presented either alone or in combination. **(B)** Schematic overview of both task conditions with specific instructions, as well as visual and acoustic secondary stimuli. Correct responses (no response in perception condition or after secondary NoGo-stimuli, responses after Go-stimuli) are marked.

Whenever the brake lights of the preceding car flashed up, participants had to press their brake pedal as fast as possible. This happened in irregular time intervals. Each flashing up of brake lights lasted for 500 ms. While driving distracting stimuli were presented, consisting of 18 country names and 18 German city names. These secondary stimuli (duration: 500 ms) were either presented acoustically via two broad-band loudspeakers by a female speaker [sound-pressure level 75 dB(A)] or visually as a 10.4 × 14 cm (4.73 by 6.36° visual angle) sign on the screen. Secondary stimuli and brake lights of the preceding car occurred either alone or concurrently in randomized order. The inter-trial-interval varied between 6 and 8 s (mean 7 s; see Figure 1A).

The task of the participants was either to ignore the secondary stimuli (perception only), or to differentially respond to these stimuli. In the latter (more demanding) discrimination condition, the participants had to respond (via key press) only to city names (Go-trials), but not to country names (NoGo-trials) or vice versa (see Figure 1B). All stimuli and stimulus combinations were presented 48 times in each task condition, with 24 Go-trials and 24 NoGo-trials in the discrimination task.

The driving session (overall duration 78 min.) was divided in six experimental blocks which were separated by short breaks. Each block contained the same stimuli and stimulus combinations, and the blocks were presented in alternating pseudo-randomized order. Before starting the driving session, the participants were informed about the task. The succession of perception and discrimination conditions was counterbalanced across the participants. The analysis of braking RTs did not reveal any overall order effects, neither in young nor in older participants (all *F* < 3.17, all *p* > 0.08).

### Data Recording

For EEG recording a “BioSemi active 2” system (BioSemi, Netherlands and United States) was used, with 32 scalp electrodes at positions of the extended international 10–20 system. Additionally, two electrodes were placed on the left and right mastoids. The EEG was sampled at 2048 Hz with amplifier bandpass 0.5 – 25.0 Hz and electrode impedance below 10 kΩ. To measure horizontal and vertical eye positions, the electro-oculography (EOG) with six additional electrodes positioned around both eyes was recorded.

### Data Analysis

#### Behavioral Data

Response times between 100 and 2,500 ms were regarded as valid answers, while responses beyond this time window were considered as omission errors. All analyses of RTs contained only correct trials. Braking RTs were separately subjected to analyses of variance (ANOVAs) with the within-subject factors TASK (perception vs. discrimination), STIMULUS [single brake light vs. brake light + secondary stimuli (Go vs. NoGo)], MODALITY (acoustic vs. visual), and the between-subject factor AGE (young vs. older). Due to the experimental design with different task conditions, there was no Go- vs. NoGo-differentiation in the perception task condition. Thus, separate analyses were performed for the perception and discrimination task (see Results).

Braking error rates were rather low and only analyzed when exceeding 2% in both age groups and conditions. Group differences of error rates were analyzed separately for different conditions by the non-parametric Mann–Whitney *U* test.

In order to test possible effects of distraction on steering performance, the lane keeping behavior was operationalized as the root-mean-squared deviance from the ideal path – overall and in specific driving segments. These segments were defined as the time windows from stimulus onset to stimulus response (for both relevant and distracting stimuli) and in the 1,500-ms time windows after stimulus response.

#### EEG Data

The raw data were digitally band-pass filtered (from 0.5 to 25 Hz; slopes 48 dB/octave) and re-referenced to the mean of the mastoid electrodes. Using the Gratton, Coles, and Donchin procedure (Gratton et al., 1983), data were corrected for ocular artifacts. With the automatic artifact rejection function in the BrainVision Analyzer software (Version 2.1; Brain Products, Gilching, Germany) all individual epochs with a maximum-minimum difference higher than 200 μV or with a maximum voltage step of 80 μV per sampling point, were excluded from further analyses.

The peaks of the P2 were defined as maximum positivity (±5 ms) in an electrode cluster of nine fronto-central electrodes around FCz (F3, Fz, F4, FC3, FCz, FC4, C3, Cz, C4) within the latency window from 100 to 300 ms after stimulus onset. The peaks of the P3b were defined as maximum positivity (±5 ms) in a cluster of nine posterior electrodes around Pz (CP3, CPz, CP4, P3, Pz, P4, PO3, POz, PO4) within the latency window from 300 to 700 after single stimulus onset, and from 500 to 900 ms after the onset of two simultaneously presented stimuli. Trials with correct answers were baseline-corrected to a 100-ms time window ending with the stimulus onset. These trials were averaged for each participant separately. EEG data (amplitude and latency of P2 at FCz and of P3b at Pz) were subjected to ANOVAs with within-subject factors TASK (perception vs. discrimination) or STIMULUS [single brake light vs. brake light + secondary stimuli (Go vs. NoGo)], and between-subject factor AGE (young vs. older).

In all behavioral and EEG data analyses, Levene’s test was used to assess the homogeneity of variance. The Greenhouse–Geisser adjustment was used to correct for violations of sphericity. In order to rank and interpret the practical significance of statistical significant results more accurately, we computed partial η^2^ as effect size.

## Results

Means and standard deviations of RTs and error rates to braking and secondary stimuli as well as P2- and P3b-amplitudes and latencies for the different task and modality conditions are provided in Table 2.

**Table 2:** Means (M) and standard deviations (SD) of braking and secondary stimulus response times and error rates, P2- and P3b-amplitudes and latencies of the two age groups in the perception and discrimination task.

	Perception				Discrimination			
		
	Young		Older		Young		Older	
				
	*M*	*SD*	*M*	*SD*	*M*	*SD*	*M*	*SD*
**Braking RT [ms]**								
Single BL	493.24	110.62	514.03	122.46	621.13	184.30	643.24	108.11
BL + acous Go	-	-	-	-	677.18	245.37	734.44	136.99
BL + acous NoGo	462.17	125.92	492.55	124.73	698.04	236.07	756.16	159.38
BL + vis Go	-	-	-	-	702.58	213.50	801.91	156.02
BL + vis NoGo	473.97	117.41	534.31	145.05	722.16	221.21	874.23	169.00
**Brake error rate [%]**								
Single BL	0.73	1.22	0.73	1.22	0.83	1.96	1.15	1.58
BL + acous Go	-	-	-	-	0.94	1.26	0.94	1.58
BL + acous NoGo	0.42	0.85	0.94	1.26	0.94	1.58	0.52	0.93
BL + vis Go	-	-	-	-	2.19	3.13	4.90	2.97
BL + vis NoGo	0.94	1.43	1.77	1.43	1.77	2.81	5.42	1.71
**Stim RT [ms]**								
Single acous Go	-	-	-	-	870.58	171.02	887.60	127.81
Single vis Go	-	-	-	-	761.26	133.24	773.00	111.74
BL + acous Go	-	-	-	-	942.52	239.54	994.46	170.81
BL + vis Go	-	-	-	-	876.88	172.96	901.38	147.54
**Stim error rate [%]**								
Single acous Go	-	-	-	-	1.35	1.55	0.42	1.09
Single vis Go	-	-	-	-	1.15	1.72	0.21	0.64
BL + acous Go	-	-	-	-	1.25	2.38	1.04	1.97
BL + vis Go	-	-	-	-	1.98	1.85	1.88	2.33
**P2-amplitude [μV]**								
Single BL	10.54	4.57	7.74	2.48	9.18	5.34	6.72	3.36
BL + acous Go	-	-	-	-	14.51	7.34	10.73	3.46
BL + acous NoGo	15.45	7.52	11.99	3.94	14.26	6.57	10.74	3.06
BL + vis Go	-	-	-	-	9.36	3.94	6.28	2.90
BL + vis NoGo	10.44	3.96	6.26	2.31	10.04	4.69	6.14	2.39
**P2-latency [ms]**								
Single BL	220.85	48.25	225.56	43.27	221.97	54.17	203.05	51.56
BL + acous Go	-	-	-	-	221.97	27.53	253.74	22.51
BL + acous NoGo	232.42	23.82	254.00	20.20	219.90	29.02	246.36	32.27
BL + vis Go	-	-	-	-	199.93	48.56	178.05	51.90
BL + vis NoGo	217.63	42.68	209.67	45.20	206.08	51.27	181.86	53.91
**P3b-amplitude [μV]**								
Single BL	12.74	4.91	10.98	3.58	12.45	4.29	8.53	3.24
BL + acous Go	-	-	-	-	9.47	3.06	5.18	2.98
BL + acous NoGo	8.23	3.67	6.64	3.96	8.20	2.81	4.57	2.50
BL + vis Go	-	-	-	-	9.16	4.30	5.12	3.13
BL + vis NoGo	7.92	4.18	6.01	3.53	8.40	3.62	5.06	3.44
**P3b-latency [ms]**								
Single BL	390.38	62.97	420.48	61.95	476.90	59.67	458.33	76.35
BL + acous Go	-	-	-	-	634.55	87.63	606.30	102.17
BL + acous NoGo	564.60	38.57	585.86	89.97	637.77	83.93	662.74	114.87
BL + vis Go	-	-	-	-	724.66	103.93	780.49	76.75
BL + vis NoGo	621.46	92.11	693.38	46.60	761.67	70.60	807.13	55.81


### Brake Responses to Single Brake Lights in an Easy vs. Difficult Task Context

In a first step we tested the effect of task context on braking responses and P3b when no secondary stimulus was present. 2 × 2-ANOVAs with within-subject factor TASK (perception, discrimination) and between-subject factor AGE (young, older) were therefore computed for single brake lights. Given that we expected P2 effects mainly in combination with secondary stimuli, this component was not analyzed here.

#### Behavioral Data

##### Response times

Response times to single brake lights were higher in the discrimination task (*M* = 632 ms, *SD* = 150) than in the perception task [*M* = 503 ms; *SD* = 116; main effect TASK: *F*(1,38) = 59.00, *p* < 0.001, η^2^ = 0.61]. There was no main effect of AGE, nor an interaction between AGE and TASK (both *F* < 0.30; both *p* > 0.05).

##### Error rates

Brake omission errors after single brake lights were rather low (<1%) in both groups, and error rates were not analyzed further.

#### P3b

##### Amplitude

**FIGURE 2:**
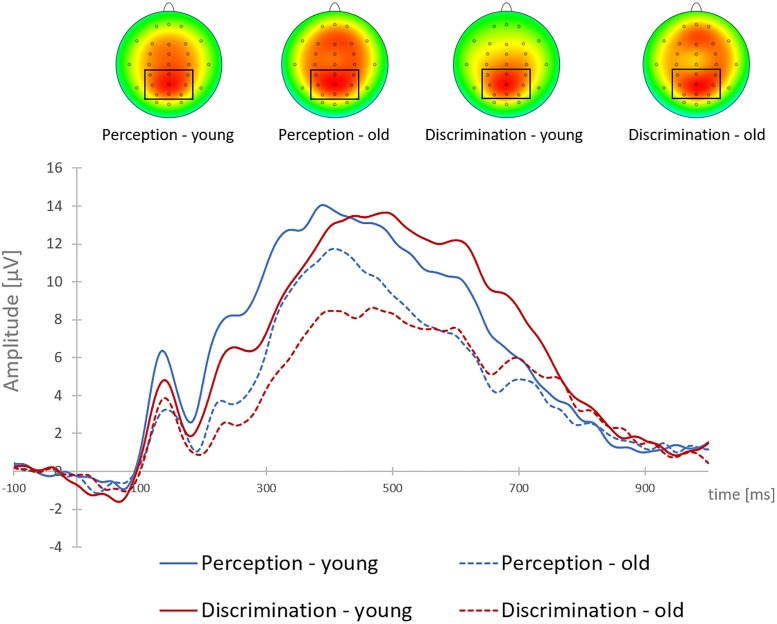
Grand-average target-locked P3b at Pz and P3b brain topographies to single brake lights, shown for young and older participants, and for the perception and discrimination tasks. The cluster of parietal electrodes for P3b analysis is marked.

The older group had overall smaller P3b-amplitudes than the younger group [old: *M* = 9.76 μV, *SD* = 3.41; young: *M* = 12.60 μV, *SD* = 4.60; main effect AGE: *F*(1,38) = 5.31, *p* = 0.027, η^2^ = 0.12]. In addition, P3b-amplitudes were smaller in the discrimination task (*M* = 10.49 μV, *SD* = 4.25) than in the perception task [*M* = 11.86, *SD* = 4.33; main effect TASK: *F*(1,38) = 14.39, *p* = 0.001, η^2^ = 0.28]. These main effects were qualified by a significant interaction of TASK × AGE [*F*(1,38) = 8.92, *p* = 0.005, η^2^ = 0.19]: *Post hoc* tests indicated that the decrease in P3b-amplitudes in the discrimination task was confined to the older group [*t*(19) = 4.72; *p* < 0.001], whereas the P3b-amplitudes of the younger group did not differ between the two tasks (*p* = 0.569; see Figure 2).

##### Latency

P3b-latency was larger in the discrimination task (*M* = 468 ms, *SD* = 69) than in the perception task (*M* = 405 ms, *SD* = 64; main effect TASK [*F*(1,38) = 17.18, *p* < 0.001, η^2^ = 0.31]. Neither the main effect of AGE nor the interaction between AGE and TASK reached significance (all *F* < 2.63, all *p* > 0.05).

To sum up, both groups omitted very few single brake lights. In the more complex (discrimination) task context braking RTs and P3b-latencies after single brake lights were in both age groups larger than in a more easy (perception) task context. In the young group, the P3b-amplitude did not differ in the two task contexts, whereas in the older group, the P3b-amplitude decreased from easy to difficult task context.

### Perception Task: Effects of Acoustic vs. Visual Secondary Stimuli

In a second step we investigated whether a simultaneously presented secondary (acoustic or visual) stimulus had an effect on brake responses when this secondary stimulus could be ignored (perception task). 3 × 2-ANOVAs with within-subject factor STIMULUS [single brake light, brake light + stimulus(acoustic), brake light + stimulus(visual)] and between-subject factor AGE (young, older) were computed.

#### Behavioral Data

##### Response times

**FIGURE 3:**
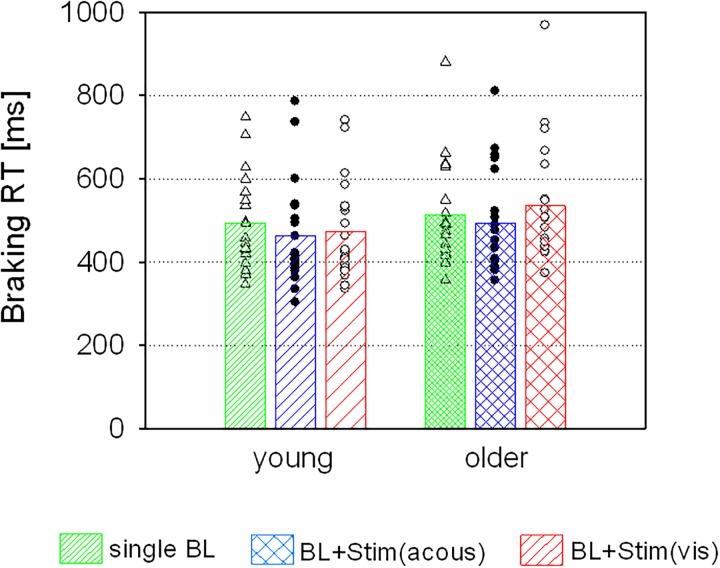
Braking RTs (means and individual values) of the two age groups in trials with single brake lights (BL), and in trials with an additional acoustic [BL + Stim(acous)] or visual stimulus [BL + Stim(vis)] in the perception task.

There was no main effect of AGE (*F* < 0.92, *p* = 0.345), but a main effect of STIMULUS [*F*(2,76) = 15.94, *p* < 0.001, η^2^ = 0.30], which was qualified by an interaction of STIMULUS and AGE [*F*(2,76) = 7.31, *p* = 0.001, η^2^ = 0.16]. *Post hoc* tests revealed an acceleration of braking RTs in trials with additional acoustic stimuli in both groups [young: *t*(19) = 3.27, *p* = 0.004; older: *t*(19) = 3.31, *p* = 0.004]. However, additional visual stimuli had different effects in the two age groups, resulting in an acceleration of braking RTs in the young group [*t*(19) = 2.85, *p* = 0.010], but a slowing in the older group [*t*(19) = -2.84, *p* = 0.010; see Figure 3].

##### Error rates

The braking error rates in the perception task were too low (<1.5%) to be analyzed further.

#### P2

##### Amplitude

**FIGURE 4:**
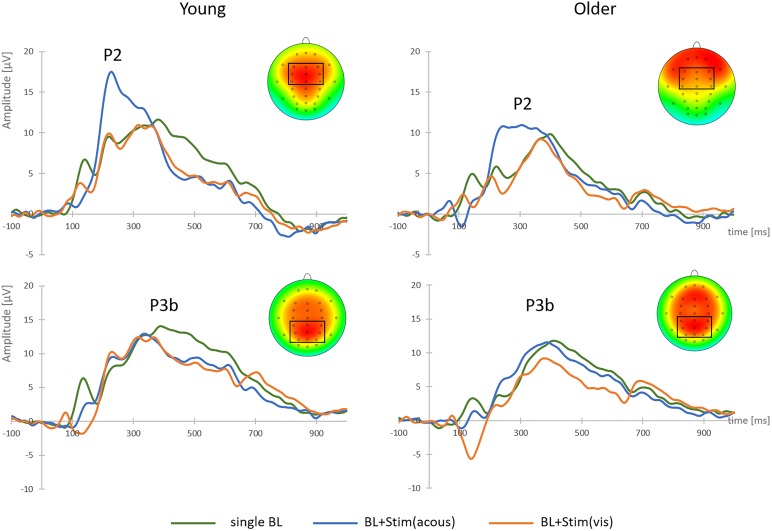
Grand-average target-locked P2 and P3b components and brain topographies to single brake lights (BL), brake lights with secondary acoustic stimulus [BL + Stim(acous)] and brake lights with secondary visual stimulus [BL + Stim(vis)] in the perception task, shown at FCz (P2) and Pz (P3b), separately for young and older participants. The clusters of fronto-central and parietal electrodes for P2 and P3b analyses are marked.

There was a main effect of STIMULUS [*F*(2,76) = 42.74, *p* < 0.001, η^2^ = 0.53]. *Post hoc* tests indicated larger P2-amplitudes after brake lights with acoustic secondary stimuli (*M* = 13.72 μV, *SD* = 6.18) relative to brake lights alone [*M* = 9.14 μV, *SD* = 3.89; *t*(39) = -7.62, *p* < 0.001] or with visual secondary stimuli [*M* = 8.35 μV, *SD* = 3.83; *t*(39) = 7.13, *p* < 0.001; see Figure 4], while there was no difference between the latter two stimulus conditions (*p* = 0.114). P2-amplitudes in general were larger in the younger than older group [young: *M* = 12.14 μV, *SD* = 5.35; old: *M* = 8.66 μV, *SD* = 2.91; main effect AGE: *F*(1,38) = 8.18, *p* = 0.007, η^2^ = 0.18], but there was no significant interaction between AGE and STIMULUS (*F* < 0.61, *p* = 0.511).

##### Latency

There were no significant main effects or interactions with AGE on P2-latency (all *F* < 2.24, all *p* > 0.05), but the main effect of STIMULUS reached significance [*F*(1,38) = 9.29, *p* < 0.001, η^2^ = 0.20]. *Post hoc* tests indicated that P2-latency of brake lights with acoustic secondary stimuli (*M* = 243 ms, *SD* = 24) were longer than with single brake lights [*M* = 223 ms, *SD* = 45; *t*(39) = -2.66, *p* = 0.011] and with visual secondary stimuli [*M* = 214 ms, *SD* = 44; *t*(39) = 3.78, *p* = 0.001], while the latter two conditions did not differ from each other (*p* = 0.110).

#### P3b

##### Amplitude

There was a main effect of STIMULUS [*F*(2,76) = 66.71, *p* < 0.001, η^2^ = 0.64]. *Post hoc* tests revealed larger P3b-amplitudes to single brake lights (*M* = 11.86 μV, *SD* = 4.33) than with visual secondary stimuli [*M* = 6.97 μV, *SD* = 3.94; *t*(39) = 7.36, *p* < 0.001] and with acoustic secondary stimuli [*M* = 7.43 μV, *SD* = 3.86, *t*(39) = 5.96, *p* < 0.001; see Figure 4], while the two latter conditions did not differ from each other (*p* = 0.172). There was no main effect or interaction with AGE on P3b-amplitude (all *F* < 2.35, all *p* > 0.05).

##### Latency

Overall, the young group showed smaller P3b-latencies (*M* = 525 ms, *SD* = 65) than the older group [*M* = 567 ms, *SD* = 66; main effect AGE: *F*(1.38) = 9.97, *p* = 0.003; η^2^ = 0.21]. Additionally, there was a significant main effect of STIMULUS [*F*(2,76) = 147.85; *p* < 0.001; η^2^ = 0.80] with shortest latencies after single brake lights (*M* = 405 ms, *SD* = 64), significantly longer latencies after brake lights with acoustic secondary stimuli [*M* = 575 ms, *SD* = 69; *t*(39) = -11.20, *p* < 0.001] and longest latencies after brake lights with visual secondary stimuli [*M* = 657 ms, *SD* = 81; relative to acoustic secondary stimuli: *t*(39) = -5.31, *p* < 0.001; relative to single brake lights: *t*(39) = -17.32, *p* < 0.001]. The interaction between STIMULUS and AGE was not significant (*F* < 1.64, *p* = 0.201).

In sum, in the perception task both groups showed shorter braking RTs, larger P2-amplitudes and longer P2-latencies in trials with secondary acoustic stimuli. In contrast, both groups showed a reduced P3b-amplitude and the longest P3b-latency in trials with secondary visual stimuli.

### Discrimination Task: Effects of Additional Go- and NoGo-Stimuli

In order to test the effects of secondary stimuli (which either had to be responded to or which had to be ignored, Go vs. NoGo) on braking performance and ERPs, 3 × 2-ANOVAs with within-subject factor STIMULUS [single brake light, brake light + stimulus(Go), brake light + stimulus(NoGo)] and between-subject factor AGE (young, older) were performed. To account for differences in the cortical processing of visual and acoustic stimuli, these ANOVAs were conducted separately for the two modalities.

#### Behavioral Data

##### Response times

**FIGURE 5:**
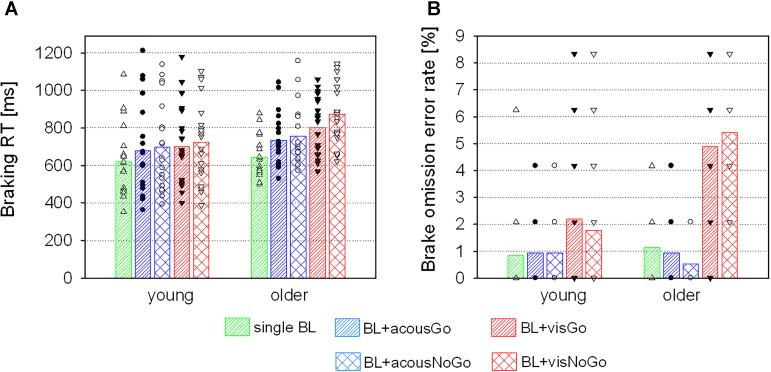
Braking RTs **(A)** and brake omission error rates **(B)** of the two age groups (means and individual values) in the discrimination task in trials with single brake lights (BL) and in trials with additional acoustic and visual Go- or NoGo-stimuli.

With acoustic secondary stimuli, a main effect of STIMULUS [*F*(2,76) = 37.80, *p* < 0.001, η^2^ = 0.50] was found, with shortest braking RTs on single brake lights (*M* = 632 ms, *SD* = 150), significantly longer braking RTs on brake lights with additional Go-stimuli [*M* = 706 ms, *SD* = 198; *t*(39) = -5.94, *p* < 0.001], and longest braking RTs on brake lights with NoGo-stimuli [*M* = 727 ms, *SD* = 201; relative to acoustic Go-stimuli: *t*(39) = -2.95, *p* = 0.005; relative to single brake lights: *t*(39) = -6.81, *p* < 0.001; see Figure 5A). There was no significant main effect or interaction with AGE (both *F* < 1.61, both *p* > 0.05).

With visual secondary stimuli, there was no main effect of AGE (*F* < 2.81, *p* = 0.102), but a main effect of STIMULUS [*F*(2,76) = 78.13, *p* < 0.001, η^2^ = 0.67], which was further qualified by an interaction of STIMULUS and AGE [*F*(2,76) = 11.36, *p* < 0.001, η^2^ = 0.23]: in the young group, relative to single brake lights braking RTs increased in the presence of additional visual stimuli [Go: *t*(19) = -5.51, *p* < 0.001; NoGo: *t*(19) = -5.66, *p* < 0.001], without any difference between visual Go- or NoGo-stimuli (*p* = 0.288). In the older group, braking RTs increased from single brake lights to brake lights with additional Go-stimuli [*t*(19) = -8.05, *p* < 0.001] and even more with NoGo-stimuli [relative to Go-stimuli: *t*(19) = -3.72, *p* = 0.001; relative to single brake lights: *t*(19) = -9.19, *p* < 0.001; see Table 2 and Figure 5A].

##### Error rates

There were no differences in braking omission rates between the two groups in trials with single brake lights or brake lights with secondary acoustic (Go or NoGo-) stimuli, according to a Mann–Whitney *U* test (all *p* > 0.05). However, there were significant group differences in trials with visual secondary Go-stimuli (*p* = 0.008) and NoGo-stimuli (*p* < 0.001), with higher error rates of older than young participants (Figure 5B).

#### P2

##### Amplitude

**FIGURE 6:**
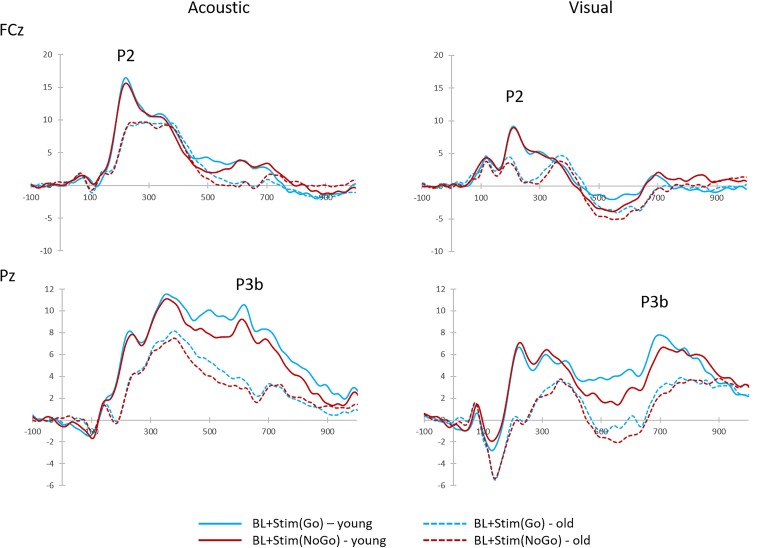
Grand-average target-locked P2 and P3b components to brake lights with secondary Go-stimulus [BL + Stim(Go)] and brake lights with secondary NoGo-stimulus [BL + Stim(NoGo)] in the discrimination task, shown at FCz (P2) and Pz (P3b), separately for acoustic and visual secondary stimuli.

With secondary acoustic stimuli, P2-amplitudes were larger in the younger (*M* = 12.65 μV, *SD* = 6.42) than the older group [*M* = 9.40 μV, *SD* = 3.29; main effect of AGE: *F*(1,38) = 4.99, *p* = 0.031, η^2^ = 0.12]. Moreover, there was a significant main effect of STIMULUS [*F*(2,76) = 36.71, *p* < 0.001, η^2^ = 0.49] with larger P2b-amplitudes to brake lights with acoustic Go-stimuli [*M* = 12.62 μV, *SD* = 5.98; *t*(39) = -6.72, *p* < 0.001] and NoGo-stimuli [*M* = 12.50 μV, *SD* = 5.36; *t*(39) = -6.90, *p* < 0.001] than to single brake lights (*M* = 7.95 μV, *SD* = 4.58 see Figure 6), while acoustic Go- and NoGo-stimuli did not differ in P2-amplitude from each other (*p* = 0.811). There was no significant interaction between AGE and STIMULUS (*F* < 0.64, *p* = 0.511).

With visual secondary stimuli, there was a main effect of AGE [*F*(1,38) = 8.37, *p* = 0.006, η^2^ = 0.18], with larger P2-amplitudes in the young group (*M* = 9.53 μV, *SD* = 4.66) than in the older group (*M* = 6.38 μV, *SD* = 2.88), but no other main effects or interactions (all *F* < 1.03, all *p* > 0.05).

##### Latency

In trials with acoustic secondary stimuli, there was no significant main effect of AGE (*F* < 2.16, *p* = 0.150), but a significant main effect of STIMULUS [*F*(2,76) = 7.34, *p* = 0.001, η^2^ = 0.16], which was further qualified by an interaction of STIMULUS and AGE [*F*(2,76) = 7.84, *p* = 0.005, η^2^ = 0.17]. *Post hoc* tests did not indicate differences in P2-latency in trials with single brake lights, brake lights with acoustic Go- and NoGo-stimuli in the younger group (all *p* > 0.370). In contrast, in the older group, there was an increase of P2-latency in trials with acoustic stimuli compared to single brake lights [relative to Go: *t*(19) = -3.82, *p* = 0.001; relative to NoGo: *t*(19) = -3.16, *p* = 0.005], but no difference between Go- and NoGo-stimuli (*p* = 0.205; see Table 2).

A different result pattern was observed in trials with visual secondary stimuli [main effect STIMULUS: *F*(2,76) = 3.98, *p* = 0.001, η^2^ = 0.10]: In both groups, Go- and NoGo-stimuli decreased P2-latency (Go: 189 ms, *SD* = 51; NoGo: 194 ms, *SD* = 53) relative to single brake lights [*M* = 213 ms, *SD* = 53; relative to Go: *t*(39) = 2.40, *p* = 0.021, relative to NoGo: *t*(39) = 2.11, *p* = 0.042], while Go- and NoGo-stimuli did not differ from each other (*p* = 0.497). There was no main effect or interaction with AGE (all *F* < 2.82, all *p* > 0.100).

#### P3b

##### Amplitude

With acoustic secondary stimuli, the P3b-amplitude was larger to single brake lights (*M* = 10.49 μV, *SD* = 4.25) than to brake lights with Go-stimuli [*M* = 7.33 μV, *SD* = 3.69; *t*(39) = 8.95, *p* < 0.001], and smallest to brake lights with NoGo-Stimuli [*M* = 6.39 μV, *SD* = 3.21; relative to single brake lights: *t*(39) = 10.88, *p* < 0.001; relative to Go-stimuli: *t*(39) = 2.66, *p* = 0.011; see Figure 6], according to a main effect of STIMULUS [*F*(1,38) = 69.57, *p* < 0.001, η^2^ = 0.65]. In addition, the P3b was larger in younger than older adults [young: *M* = 10.04 μV, *SD* = 3.39; old: *M* = 6.09 μV, *SD* = 2.91; main effect AGE: *F*(1,38) = 18.43, *p* < 0.001, η^2^ = 0.33). An interaction between AGE and stimulus did not reach significance (*p* = 0.660).

Relative to single brake lights, the P3b-amplitude with visual secondary stimuli was decreased in the presence of Go-stimuli [*M* = 7.14 μV, *SD* = 4.24; *t*(39) = 8.52, *p* < 0.001] and NoGo-stimuli [*M* = 6.73 μV, *SD* = 3.87; *t*(39) = 8.43, *p* < 0.001], according to a main effect of STIMULUS [*F*(2,76) = 51.41, *p* < 0.001, η^2^ = 0.58]. However, in contrast to the acoustic modality, the P3b reduction did not differ between Go- and NoGo-stimuli (*p* = 0.269). Again, there was a main effect of AGE [*F*(1,38) = 12.35, *p* = 0.001, η^2^ = 0.25; young: *M* = 10.00 μV, *SD* = 4.07; older: *M* = 6.24 μV, *SD* = 3.27], but no interaction between AGE and STIMULUS (*p* = 0.645).

##### Latency

With auditory secondary stimuli, there was a main effect of STIMULUS [*F*(2,76) = 45.54, *p* < 0.001, η^2^ = 0.55]. *Post hoc* tests showed that the P3b-latency (single brake lights: *M* = 467 ms, *SD* = 68) increased with additional acoustic stimuli for both Go- or NoGo-stimuli [Go: *M* = 620 ms, *SD* = 95; *t*(39) = -7.83, *p* < 0.001; NoGo: *M* = 650 ms, *SD* = 100; *t*(39) = -8.33, *p* < 0.001], while Go- and NoGo-trials did not differ from each other (*p* > 0.05). Main effects or interactions with AGE did not occur (all *F* < 0.96, all *p* > 0.05).

With visual secondary stimuli, there were no main effect of AGE (*p* = 0.107), but of STIMULUS [*F*(2,76) = 279.18, *p* < 0.001, η^2^ = 0.88], which was qualified by an interaction of STIMULUS and AGE [*F*(2,76) = 3.73, *p* = 0.033, η^2^ = 0.09]. In both age groups, P3b-latency increased from single brake lights to brake lights with Go-stimulus [young: *t*(19) = -10.13, *p* < 0.001; older: *t*(19) = -13.71, *p* < 0.001] and NoGo-stimuli [young: *t*(19) = -14.65, *p* < 0.001; older: *t*(19) = -17.63, *p* < 0.001; see Table 2]. The increase of P3b-latency across stimulus conditions was larger in the older group.

To sum up, in the discrimination task, the braking omission error rate of the older participants was higher than that of the young ones only in trials with secondary visual stimuli. NoGo-stimuli were associated with longer braking RTs. In trials with acoustic stimuli this was observed in both groups, in trials with visual stimuli only in the older group. Secondary acoustic stimuli increased the P2-amplitude in both groups, as well as the P2-latency in the older group. In contrast, P2-latency decreased with visual secondary stimuli in both groups. Young and older participants showed smaller P3b-amplitudes and longer P3b-latencies in trials with (any) secondary stimuli. This effect was enhanced in trials with visual secondary stimuli and in older participants.

### Discrimination Task: Effects of Secondary Stimulus’ Type and Modality

In a final analysis we tested whether acoustic and visual secondary Go- and NoGo- stimuli differed in their effects on braking behavior and ERP measures. 2 × 2 × 2 ANOVAs were conducted with within-subject factors STIMULUS [brake light + stimulus(Go) vs. brake light + stimulus(NoGo)] and MODALITY (acoustic vs. visual) and between-subject factor AGE (young vs. older).

#### Behavioral Data

##### Response times

In addition to main effects of STIMULUS [*F*(1,38) = 15.41, *p* < 0.001, η^2^ = 0.29] and MODALITY [*F*(1,38) = 27.75, *p* < 0.001, η^2^ = 0.42], there was a two-way interaction of MODALITY and AGE [*F*(1,38) = 9.29, *p* = 0.004, η^2^ = 0.20] and a three-way interaction of STIMULUS, MODALITY, and AGE [*F*(1,38) = 4.13, *p* = 0.049, η^2^ = 0.10]. *Post hoc* tests revealed that braking RTs in young adults increased from brake lights with additional acoustic Go-stimuli to acoustic NoGo-stimuli [*t*(19) = -2.22, *p* = 0.039], and further to visual NoGo-stimuli [*t*(19) = -2.24, *p* = 0.038]. In older adults, braking RTs with visual stimuli were larger than with acoustic stimuli [Go: *t*(19) = -3.09, *p* = 0.006; NoGo: *t*(19) = -5.40, *p* < 0.001], and larger with visual NoGo-stimuli than with visual Go-stimuli [*t*(19) = -3.72, *p* = 0.001], while the difference between acoustic Go- and NoGo-stimuli did not reach statistical significance (*p* = 0.067; see Table 2 and Figure 6). There was no main effect of AGE and no interaction between MODALITY and STIMULUS (all *F* < 3.73, all *p* > 0.05).

##### Error rates

Mann-Whitney *U* tests of the braking omission rates in trials with secondary stimuli of different modalities indicated significant group differences in trials with visual secondary Go-stimuli (*p* = 0.008) and NoGo-stimuli (*p* < 0.001), with higher error rates of older than young participants (as already described in Subsection “Behavioral Data” of Section “Discrimination Task: Effects of Additional Go- and NoGo-Stimuli”; Figure 5B).

#### P2

##### Amplitude

Young adults showed larger P2-amplitudes (*M* = 11.47 μV, *SD* = 5.58) than older adults [*M* = 8.12 μV, *SD* = 3.03; main effect AGE: *F*(1,38) = 8.64, *p* = 0.006, η^2^ = 0.19]. In trials with acoustic secondary stimuli, P2-amplitudes were larger (*M* = 12.56 μV, *SD* = 5.11) than with visual secondary stimuli [*M* = 7.96 μV, *SD* = 3.48; main effect MODALITY: *F*(1,38) = 45.40, *p* < 0.001, η^2^ = 0.54]. The main effect of STIMULUS or any interaction did not reach significance (all *F* < 0.79, all *p* > 0.05).

##### Latency

There was a main effect of MODALITY [*F*(1,38) = 31.75, *p* < 0.001, η^2^ = 0.46] that was qualified by an interaction of MODALITY and AGE [*F*(1,38) = 11.15, *p* = 0.002, η^2^ = 0.23]. *Post hoc* tests showed that only in older adults P2-latencies were larger with secondary acoustic (*M* = 250 ms, *SD* = 25) than visual stimuli [*M* = 203 ms, *SD* = 49; *t*(19) = 5.98, *p* < 0.001], while there was no such modality effect in young adults (*p* = 0.099). There was no significant main effect of AGE or STIMULUS nor any other significant interaction (all *F* < 1.72, all *p* > 0.197).

#### P3b

##### Amplitude

P3b-amplitudes did not differ between stimulus MODALITY (*p* = 0.807), but were larger in younger than older adults [young: *M* = 8.81 μV, *SD* = 3.45, older: *M* = 4.98 μV, *SD* = 3.01; main effect AGE: *F*(1,38) = 17.46, *p* < 0.001, η^2^ = 0.32] and with Go- than NoGo-stimuli [Go: *M* = 7.23 μV, *SD* = 3.37, NoGo: *M* = 6.56 μV, *SD* = 3.03; main effect STIMULUS: *F*(1,38) = 5.60, *p* = 0.023, η^2^ = 0.13, see Figure 6). There were no interactions (all *F* < 1.43, all *p* > 0.05).

##### Latency

There were main effects of STIMULUS [*F*(1,38) = 5.46, *p* = 0.025, η^2^ = 0.13] with longer latencies after NoGo-stimuli (*M* = 717 ms, *SD* = 81) than Go-stimuli (*M* = 687 ms, *SD* = 93), and MODALITY [*F*(1,38) = 98.14, *p* < 0.001, η^2^ = 0.72] with longer latencies in trials with visual secondary stimuli (*M* = 768 ms, *SD* = 77) than acoustic secondary stimuli (*M* = 635 ms, *SD* = 97). Neither the main effect of AGE nor any interaction reached significance (all *F* < 3.79, all *p* > 0.05).

To sum up, young and older participants showed the largest braking RTs in trials with visual NoGo-stimuli. Both groups showed larger P2-amplitudes, and the older group also larger P2-latencies, in trials with acoustic secondary stimuli than with visual secondary stimuli. Moreover, both groups also showed larger P3b-amplitudes and shorter P3b-latencies with Go- than in NoGo-stimuli as well as longer P3b-latencies in trials with visual than acoustic secondary stimuli.

### Responses to the Secondary Stimuli and Lane-Keeping Behavior

In order to analyze the responses to the secondary stimuli, 2 × 2 × 2 ANOVAs of stimulus RTs were conducted with within-subject factors STIMULUS (secondary stimulus alone vs. brake light + secondary stimulus) and MODALITY (acoustic vs. visual), and between-subjects factor AGE (young vs. older). Given the low error rates (omission errors in Go-trials and false alarms in NoGo-trials) across age groups and conditions, these error rates were not analyzed further.

#### Response Times

Response times to the secondary stimuli were larger in combination with brake lights (*M* = 929 ms, *SD* = 183) than alone [*M* = 823 ms, *SD* = 135; main effect STIMULUS: *F*(1,38) = 65.41, *p* < 0.001, η^2^ = 0.63], and larger with acoustic stimuli (*M* = 924 ms, *SD* = 178) than visual stimuli [*M* = 828 ms, *SD* = 140; main effect MODALITY; *F*(1,38) = 37.90, *p* < 0.001, η^2^ = 0.50]. There were no effects of AGE or any interactions (all *F* < 3.50, all *p* > 0.05).

The analysis of lane-keeping performance did not indicate a significant main effect of AGE, neither in overall lane-keeping performance (*p* = 0.427), nor in specific driving segments (all *F* < 1.89, all *p* > 0.176). Moreover, there were no significant interactions of AGE with STIMULUS, TASK, or MODALITY (all *F* < 3.60, all *p* > 0.065).

## Discussion

The present study examined the effect of acoustic and visual distraction on brake response behavior in younger and older drivers. There were no differences in primary task performance (lane keeping) between the two age groups, but they differed in their braking responses depending on task and distraction conditions. These differences were reflected on the behavioral level by larger braking RTs and higher braking error rates, and on the neurophysiological level by modifications in the cortical processing of the brake lights.

### Effects of Task Context

There were no differences between the two age groups in braking RTs after single brake lights without any secondary stimulus, neither in the perception, nor the discrimination task. Interestingly, in both groups braking RT increased in the discrimination task, although the stimulus itself (i.e., the brake lights) and what the drivers should do with it (i.e., pressing the brake pedal) did not changed. This result is in line with another study in which effects of performance expectancies on actual performance have been reported (Reinhard and Dickhäuser, 2009). Thus, it appears that the very announcement of the more difficult discrimination condition and (with it) the anticipation of the possible occurrence of an additional imperative stimulus led to a partition of cognitive resources between the primary and secondary task. This assumption of a preventive resource allocation to the potential secondary stimulus is also reflected by ERP measures, indicating increased P3b-latencies and (in the older group) smaller P3b-amplitudes to single brake lights in the discrimination task. Considering driving in real traffic, this suggested that even the anticipation or announcement of a difficult driving task or driving situation may decrease the responses to relevant stimuli. This could result in the (conscious or unconscious) use of compensation strategies and self-regulation driving behavior, which has been often reported for driving in real traffic (e.g., Molnar and Eby, 2008; Donorfio et al., 2009), but also in driving simulation studies (e.g., Wechsler et al., 2018) and other laboratory tasks in the driving context (e.g., Feng et al., 2018). Potential compensatory strategies in difficult driving situations in reality could be the reduction of driving speed, the increase of the distance to preceding cars, and also the deliberate turning off of infotainment systems.

### Effects of Additional Acoustic and Visual Stimuli in Perception Task

In the perception task used here, all secondary stimuli could to be ignored. Nevertheless, secondary acoustic stimuli presented simultaneously to the brake lights led to faster braking responses in both age groups. This acceleration effect could be related to an alerting function of acoustic stimulation, which was on a neurophysiological level indicated by a more pronounced P2 amplitude. In addition, the P2 has been related to processes of sensory gating, selective attention, and feature detection (O’Donnell et al., 1997; Crowley and Colrain, 2004; Potts, 2004; Lijffijt et al., 2009) as well as to protective mechanisms against interference from irrelevant stimuli (García-Larrea et al., 1992; Benikos et al., 2013). The increase in P2 amplitude observed here could indicate that these early stages of processing are enhanced by the additional acoustic stimulus. Enhanced responses to multisensory signals have also been reported in previous studies in young and (even more) in older adults (e.g., Peiffer et al., 2007). Faster braking responses in the presence of secondary acoustic stimuli are also in line with the multiple resource theory of Wickens (2002, 2008), assuming that interference effects should be less pronounced when stimuli are of different modalities.

In contrast to acoustic stimuli, the effect of visual secondary stimuli clearly differed between the two age groups: In the young group, visual stimuli also led to decreased braking RTs (and slightly more brake errors), while in the older group the presence of additional visual stimuli results in an increase in braking RTs and in higher error rates. In both groups, visual secondary stimuli were associated with a smaller P3b amplitude, reflecting reduced processing of the brake lights.

This obvious distinction in the way younger and older adults managed the processing of acoustic and visual distractors could indicate age-related differences in inhibitory subprocesses. As proposed by some authors, these subprocesses comprise the three factors of (1) inhibition of prepotent (i.e., dominant) responses, (2) resistance to distractor interference and (3) resistance to proactive interference (Friedman and Miyake, 2004; Pettigrew and Martin, 2014; Stahl et al., 2014; see Rey-Mermet et al. (2017) for an overview of other taxonomies about inhibition). Rey-Mermet et al. (2017) analyzed the performance of young and older participants in 11 laboratory tasks that are typically used to measure inhibition. They found worse performance of older adults only in the tasks that are associated with the inhibition of prepotent responses, but even better performance of the older group in tasks associated with resistance to distracter interference. However, the authors mentioned a relatively low explanatory power of the model – potentially because stimulus structures and materials used in these tasks were quite divergent. In the present study, the same secondary acoustic and visual stimuli with different task instructions were used to modulate task workload and stimulus modality. The two resulting tasks may therefore be regarded as reflecting the resistance to distractor interference (perception task) and the inhibition of prepotent responses (discrimination task). The effects of secondary stimuli in the (easy) perception task may thus be an indicator for an impaired resistance to distractor interference in older adults. However, this applies only to distractors of the same modality that are assumed to use the same mental resources.

### Effects of Secondary Acoustic and Visual Go- and NoGo-Stimuli in Discrimination Task

In the discrimination task, participants had to distinguish relevant stimuli from irrelevant ones and respond to the relevant stimuli, while inhibiting the response to the irrelevant ones. When these secondary stimuli occurred in combination with the brake lights, the task became a dual task. The simultaneous performance of two tasks usually results in a performance decrease in at least one of the two tasks. This effect is typically enhanced with increasing age (Verhaeghen et al., 2003).

Impairment of dual task performance can be attributed to sensory, motor, or cognitive interference with quite different performance pattern each (e.g., Pashler, 1994; Wild-Wall et al., 2011; Strayer et al., 2016; Wechsler et al., 2018). In the task used in the present study, sensory interference should lead to higher braking RT in trials with two stimuli (brake light plus secondary stimulus) compared to single brake lights, without any differences between Go- and NoGo-trials. Expecting motor interference, the additional motor response in Go-trials should result in increased braking RT. In contrast, expecting cognitive interference or, more precisely, problems in the inhibition of prepotent responses, there should be an increase of braking RT in NoGo-trials compared to Go-trials.

The present results of the difficult discrimination task indicated that the benefit of separate stimulus modalities and resources used for the processing of acoustic stimuli diminished in both groups: In trials in which participants had to respond to additional acoustic stimuli (Go), braking RTs increased. This increase was even more pronounced in trials, in which participants did not have to respond to them (NoGo). Thus, the inhibition of prepotent responses seems to be more challenging under this condition. Importantly, braking response performance of young and older adults were only comparable as long as the additional stimuli were presented in a different modality. However, differences between the two age groups became manifest with additional visual stimuli, resulting in significantly higher braking RTs after NoGo-stimuli compared to Go-stimuli in older adults. The increase in brake omission errors due to visual secondary stimuli in the older group was even more dramatic (cf. Figure 5B). In sum, this pattern of results suggests an impaired inhibition of prepotent responses in older adults, when stimuli of the same modality are present. The co-occurrence of impaired resistance to distractor interference as well as impaired inhibition of prepotent responses in older adults is in line with other studies, that found a strong correlation between these two inhibition subforms (Friedman and Miyake, 2004; Pettigrew and Martin, 2014).

The behavioral results were associated with modulations of ERPs. On the one hand, the P2 was stronger when the brake lights were combined with secondary acoustic stimuli. This effect was also observed in the perception task and might be related to alerting effects and enhanced early stimulus processing. However, different from the perception condition, the increase in P2 in the discrimination task was not associated with enhanced braking performance. Importantly, the P2 increase did not depend on whether the secondary stimulus required a Go- or NoGo-response, suggesting that the P2 effect was independent from later processes of stimulus evaluation and inhibition of prepotent responses. In contrast to the P2, the P3b-amplitude decreased and the P3b-latency increased in the presence of secondary stimuli. These effects were more pronounced with NoGo- than Go-stimuli, and especially so in older adults. The generally smaller P3b-amplitude of the older group compared to the young group is in line with the literature and has been associated with reduced availability of attentional resources (e.g., Gajewski and Falkenstein, 2014; Gaal and Czigler, 2015; Enriquez-Geppert and Barceló, 2018). As the P3b reflects controlled cognitive attentional and stimulus evaluation processes (for review, see Polich, 2007), the P3b modulations observed here are thus the correlates of the negative effects of secondary stimuli, especially on the later processing of the brake lights.

### Limitations

There is an ongoing debate about the general transferability of driving simulator performance to real driving (e.g., Caird and Horrey, 2011; Karthaus and Falkenstein, 2016) and some evidence suggests that effects of distraction on RTs are rather be underestimated than overestimated in laboratory settings (Caird et al., 2018). However, there are some differences between distracted driving in the driving simulator and on the road: First of all, a monotonous grassland may not be a very representative driving scenario for rural and even less representative for urban areas. But it can be assumed that the distracting effects of acoustic or visual secondary stimuli will even be enhanced in more complex driving scenarios. However, further research is needed to analyze area-specific distraction in different age groups. Second, in the experimental setting, there are usually clear instructions indicating which stimuli have to be responded to and which have to be ignored. In real driving, drivers have to decide this by themselves. This decision depends on the drivers’ own characteristics and current situational conditions (Collet et al., 2010) and can itself be affected by communication-based distraction leading to safety-related misjudgments (Cooper and Zheng, 2002). Especially older adults often adapt their driving behavior to compensate sensory, motor, or cognitive deficits, e.g., by slowing down in complex situations. In the present experimental setting, an adjustment of speed was not possible. However, the results of previous studies on compensatory behavior in driving are mixed (McCartt et al., 2006; Choudhary and Velaga, 2017; Caird et al., 2018) and could not confirm the automatical or mandatory use of compensation strategies in driving.

## Conclusion

Anticipation of high demanding tasks – even without presence of any distracting stimuli – affected braking performance in both age groups. However, the potential effect of resource partition in difficult tasks was more pronounced in older adults, suggesting an impaired resistance to distractor interference and reduced inhibition of prepotent responses, especially when secondary irrelevant stimuli and relevant stimuli share the same modality. Age-related deficits in cognitive control (and in inhibition in particular) differed between the inhibitory subprocesses required, depending on task workload and stimulus modality. These findings have implications for the traffic safety of older drivers: On the one hand, older drivers should reduce driving-irrelevant stimulation as much as possible (for example, by switching off non-necessary car information systems). On the other hand, unavoidable secondary information (for example, from navigation systems) should better be provided acoustically than visually, and not simultaneously to safety-related information.

## Data Availability Statement

The raw data supporting the conclusions of this manuscript will be made available by the authors, without undue reservation, to any qualified researcher.

## Ethics Statement

This study was carried out in accordance with the recommendations of the local ethics committee with written informed consent from all subjects. All subjects gave written informed consent in accordance with the Declaration of Helsinki. The protocol was approved by the local ethics committee.

## Author Contributions

MK, SG, and EW developed the design of the study. MK was responsible for data recording and analyzing. MK and SG wrote the manuscript in consultation with EW, who aided in interpreting the data. All authors discussed the results and contributed to the final manuscript.

## Conflict of Interest Statement

The authors declare that the research was conducted in the absence of any commercial or financial relationships that could be construed as a potential conflict of interest.
